# Advancements in Flame-Retardant Systems for Rigid Polyurethane Foam

**DOI:** 10.3390/molecules28227549

**Published:** 2023-11-11

**Authors:** Yao Yuan, Weiliang Lin, Yi Xiao, Bin Yu, Wei Wang

**Affiliations:** 1 Fujian Provincial Key Laboratory of Functional Materials and Applications, School of Materials Science and Engineering, Xiamen University of Technology, Xiamen 361024, China; weilianglin318315@163.com (W.L.); ahxiaoyi@163.com (Y.X.); 2 State Key Laboratory of Fire Science, University of Science and Technology of China, Hefei 230026, China; yubin@ustc.edu.cn; 3 School of Mechanical and Manufacturing Engineering, University of New South Wales, Sydney, NSW 2052, Australia

**Keywords:** rigid polyurethane foam, flame retardancy, smoke toxicity suppression, flame retardants

## Abstract

The amplified employment of rigid polyurethane foam (RPUF) has accentuated the importance of its flame-retardant properties in stimulating demand. Thus, a compelling research report is essential to scrutinize the recent progression in the field of the flame retardancy and smoke toxicity reduction of RPUF. This comprehensive analysis delves into the conventional and innovative trends in flame-retardant (FR) systems, comprising reactive-type FRs, additive-type FRs, inorganic nanoparticles, and protective coatings for flame resistance, and summarizes their impacts on the thermal stability, mechanical properties, and smoke toxicity suppression of the resultant foams. Nevertheless, there are still several challenges that require attention, such as the migration of additives, the insufficient interfacial compatibility between flame-retardant polyols or flame retardants and the RPUF matrix, and the complexity of achieving both flame retardancy and mechanical properties simultaneously. Moreover, future research should focus on utilizing functionalized precursors and developing biodegradable RPUF to promote sustainability and to expand the applications of polyurethane foam.

## 1. Introduction

Rigid polyurethane foam (RPUF) is a highly adaptable type of polymer foam that finds utility across a diverse array of industries and everyday household applications, such as building insulation and the structural portions of roofs, walls, floors, and furniture, primarily due to its excellent insulation properties, durability, lightweight nature, and flexibility in customization [1,2,3,4,5,6,7]. According to a recent report, the worldwide polyurethane (PU) market is predicted to attain a value of USD 88 million by the year 2026, exhibiting a compound annual growth rate (CAGR) of 6.0% [8]. It is evident from the data that the number of scientific articles related to PU and PU foam has more than doubled during the period of 2010–2020 compared to 1996–2009, serving as a clear indication. The primary usage of PU in the market is in PU foam, which accounts for up to 67% of the world’s total foam consumption [9].

Rigid polyurethane foam is a type of foam insulation made by combining two liquid components, a polyol (a type of alcohol) and an isocyanate (a type of chemical compound), which react and expand to form a solid foam material [10,11,12,13]. In regard to flammability, the cellular structure and organic composition of RPUF make it combustible and susceptible to burning upon exposure to high temperatures or flames [14,15,16,17]. Moreover, the combustion of RPUF may result in the emission of toxic smoke and gases, such as carbon monoxide (CO), nitrous oxides (NO_x_), and hydrogen cyanide (HCN), which can be hazardous to human health [18]. For this reason, it is crucial to explore flame-retardant RPUF and to expand its practical applications [5,19,20,21,22,23].

There are three primary approaches being utilized to address the limitations of RPUF, as depicted in Table 1. The first method is copolymerization, which entails the chemical modification of the surface and core of the polymer [24,25]. The final two methods involve adding flame-retardant (FR) additives to the polymer matrix through mixing or coating [5,21,26,27,28,29]. Meanwhile, the first option has been demonstrated to be the durable and feasible approach for pristine RPUF. These flame retardants are primarily composed of organic compounds which possess a flame-retardant segment that can create covalent bonds with RPUF [30]. As a result, the integration of flame retardants into RPUF composites can lead to a considerable improvement in the compatibility between the polymer and the flame retardants as well as provide additional benefits such as enhanced mechanical properties, improved thermal stability, and better compatibility with other additives [31,32].

Numerous reviews have been published regarding the utilization of additive-type flame retardants to enhance the flame resistance of RPUF, and this has been established as the most practical and economical approach for untreated RPUF [28,33,34]. Phosphorus-based and nitrogen-based flame retardants are among the most frequently utilized additives in the fabrication of RPUF composites [35]. Phosphorus-based and nitrogen-based FRs are the most used FRs in RPUF composites. Phosphorus-based FRs, such as ammonium polyphosphate (APP) and melamine polyphosphate (MPP), have excellent flame-retardant properties and generate char upon exposure to heat, which further protects the polymer matrix from combustion [33,36]. Nitrogen-based FRs, such as melamine cyanurate (MC) and guanidine phosphate (GP), generate nonflammable gases and effectively suppress the flames during combustion. Furthermore, the use of nanocomposites that contain nanofillers, like layered double hydroxides (LDHs) and graphene oxide (GO), has demonstrated potential as effective flame retardants and smoke suppressants for RPUF composites [37,38]. These nanofillers have a large surface-area-to-volume ratio, improving their ability to interact with the polymer matrix and impede the spread of flames.

The growing utilization of RPUF has intensified the significance of its flame-retardant properties in driving demand. Consequently, an imperative research report is warranted to investigate recent advancements in the field of the flame retardancy and smoke toxicity suppression of RPUF, given the limited extant literature on this topic. This comprehensive review centers on conventional and emerging trends in flame-retardant (FR) systems, including reactive-type and additive-type FRs, inorganic nanoparticles, and protective coatings, aimed at enhancing the flame retardancy and smoke toxicity suppression of RPUF. This review also covers an overview of the preparation and properties of RPUF and the current trends in flame-retardant strategies for RPUF and a discussion on the future outlook of flame-retardant RPUF.

**Table 1:** Examples of RPUF composites with various flame retardants.

Flame-Retardant Type	Composite	Remarks	Ref.
Reactive flame retardants	RPUF/AMPO (polyol-bis(hydroxymethyl)-N, N-bis(2-hydroxyethyl)aminomethylphosphine oxide)	- The incorporation of 10.5 wt.% AMPO resulted in an increase in the LOI value from 20.0% in the pure RPUF to 23.4%. - However, the inclusion of AMPO in RPUF resulted in a 21.2% decrease in compressive strength.	[39] (1982)
	RPUF/GEP (glycerol/ethanol phosphate)	- The incorporation of 8.0 wt.% glycerol/ethanol phosphate (GEP) resulted in an increase in the LOI of RPUF to 23.5% - However, the total heat release (THR) and peak smoke production rate (PSPR) were increased by 52.0% and 170.0%.	[40] (2015)
	RPUF/PPGE (phenylphosphoryl glycol ether oligomer)	- By incorporating 10.0 wt.% of the additive, RPUF demonstrated an LOI of 24.5%, obtained a UL-94 V-1 rating, and experienced a notable 1.5% enhancement in compressive strength.	[41] (2019)
Additive flame retardants	RPUF/pEG-P(MA) (pulverized expandable graphite (pEG)-poly(methyl methacrylate-acrylic acid) copolymer)	- The RPUF containing 10 wt.% of flame-retardant particles exhibited excellent flame retardancy, as evidenced by a high LOI of 26 vol.%. - The RPUF/pEG-P(MA) composites demonstrated a compressive modulus of 48.4 MPa and a compressive strength of 2.8 MPa.	[42] (2011)
	RPUF/MATMP (melamine amino trimethylene phosphate)	- The incorporation of 15.0 wt.% of MATMP in RPUF resulted in a UL-94 V-0 rating accompanied by a 34.0% decrease in PHRR and an LOI of 25.5%. - At lower loading levels (<10.0 wt.%), MATMP simultaneously improved the compressive strength and thermal insulating properties of RPUF.	[43] (2017)
	RPUF/MFAPP (melamine–formaldehyde resin-microencapsulated ammonium polyphosphate)	- RPUF/MFAPP30 attained a V-1 rating in the UL-94 test, exhibiting an LOI of 21.3 vol.% compared to RPUF/APP30 with an equal load of APP. - The RPUF/MFAPP30 demonstrated a compressive strength of 0.295 MPa, exhibiting a 13.5% increase compared to RPUF/APP30.	[44] (2020)
Flame-retardant coating	RPUF/alginate/clay aerogel	- The RPUF coated with alginate/clay aerogel exhibited an impressive LOI of 60.0%, with 32.0% and 37.0% reductions in PHRR and TSR as compared to the untreated foam. - However, the presence of the aerogel within the porous foam noticeably compromised its thermal conductivity.	[45] (2016)
	RPUF/poly(VS-co-HEA) (copolymerization of hydroxyethyl acrylate (HEA) and sodium vinylsulfonate (VS))	- The resulting RPUF obtained a UL-94 V-0 rating; an LOI of 35.5%; and significant reductions of 87.0% and 71.0% in PHRR and TSR, respectively, compared to the untreated foam. - The introduction of HGM to the coating enabled the coated PU foam to maintain a low thermal conductivity.	[2] (2021)

## 2. Reactive-Type Flame Retardants

**Figure 1:**
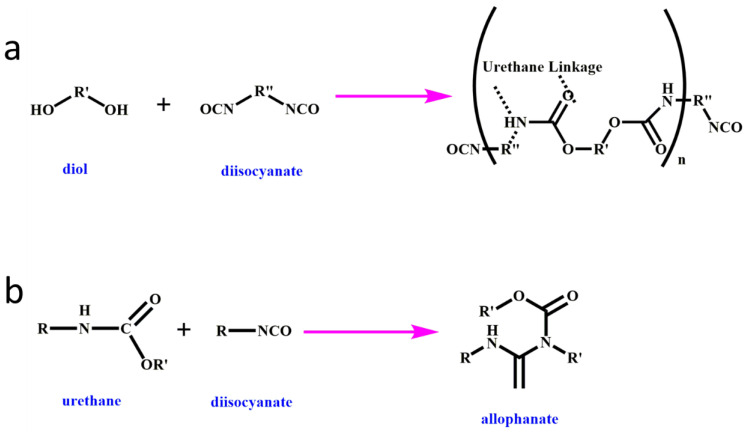
Basic reaction scheme for (**a**) urethane and (**b**) allophanate formation.

The process of forming RPUF involves various reactions, including urethane formation, crosslinking reactions, and foaming reactions facilitated using a chemical blowing agent. The formation of the urethane linkage is illustrated in Figure 1a, while Figure 1b shows how the urethane group reacts with an isocyanate group to create allophanate, which results in chemical crosslinking. Reactive flame retardants demonstrate favorable interfacial compatibility with the matrix due to their chemical bonding interactions, resulting in minimal impact on the mechanical properties of RPUF. Meanwhile, reactive flame retardants containing multiple hydroxyl, amino, or epoxy groups can serve as polyols in the curing process of RPUF, providing flame retardant properties through the presence of phosphorus, nitrogen, or sulfur elements in their structure [8,41,46,47,48]. Additionally, reactive flame retardants with chemical bonding interactions are highly durable in industrial applications, as they prevent migration from the RPUF matrix. This section focuses on recent advancements in reactive flame retardants.

### 2.1. Incorporation of Phosphorus-Containing Groups

Phosphorous-containing flame-retardant polyols are a type of reactive flame retardants, which can be used as a substitute for conventional polyols in the preparation of RPUF to enhance its flame-retardant properties [41,49]. The effectiveness of phosphorus-based flame retardants in minimizing the flammability of polyurethane foam has been well established, leading to its widespread adoption in industries ranging from construction and transportation to electronics [32,50]. Polyols containing phosphorus-based flame retardants possess multiple hydroxyl groups that can actively engage in the curing reaction of polyurethane foam. The incorporation of phosphorus-containing flame retardants in polyols enables the formation of chars and reduces the release of flammable gases during combustion [51].

**Figure 2:**
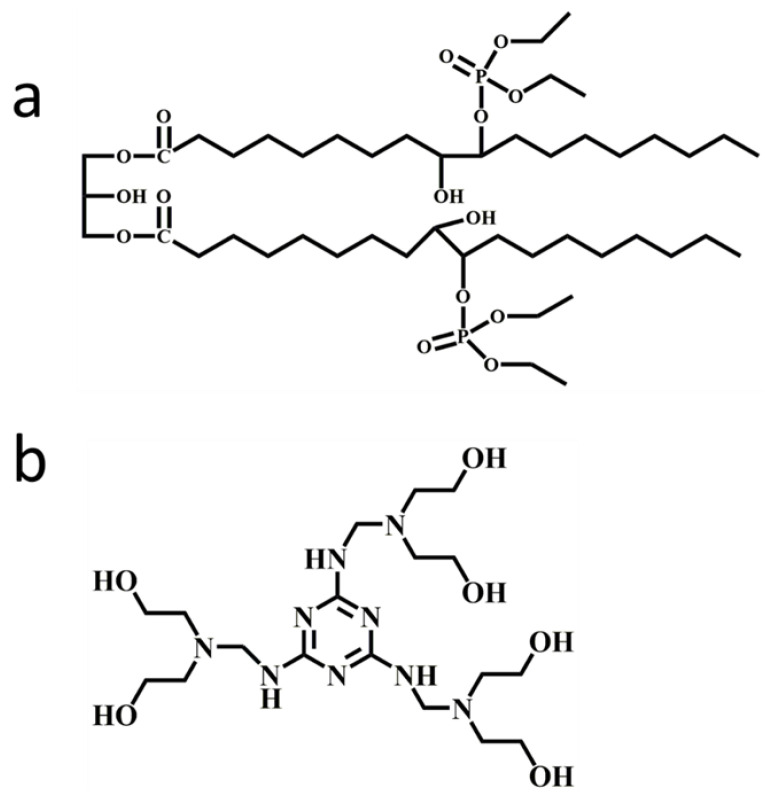
Structure of different flame retardants: (**a**) COFPL and (**b**) MADP.

One of the studies in this area focused on the application of biobased flame-retardant polyols. In their study, Bhoyate et al. [52] explored the fire safety of a polyol sourced from limonene, which was chemically modified with phenyl phosphonic acid. According to their findings, adding 1.5 wt.% of phosphorus through chemical modification could decrease the self-extinguishing time from 81 s to 11.2 s. Zhang et al. [53] employed the approach illustrated in Figure 2a to produce castor oil phosphate flame-retardant polyol (COFPL), a flame-retardant polyol derived by incorporating a phosphate group into the polyol. The process involves the preparation of glycerolized castor oil (GCO) and the epoxidation of GCO, which is ultimately converted to COFPL through a reaction with diethyl phosphate. Wang et al. [54] developed two biobased flame-retardant polyols (CODEOA and CPPA) from a modified vegetable oil. By incorporating epoxidized polyols (BIO_2_) and derived carbon materials into the RPUF matrix, the heat release rate (HRR), total heat release (THR), and total smoke production (TSP) can be significantly reduced.

Currently, green materials such as plant oil, tung oil, and lignin have been chosen as the primary synthetic resources. However, the flame-retardant performance of biobased polyols is hindered by their long-chain structure, resulting in the low content of flame-retardant elements (phosphorus, nitrogen, silicon, etc.). Zhou et al. [55] synthesized tung-oil-based polyols through the ring-opening reaction of epoxidized tung oil and silane-coupling agents. Although the limiting oxygen index of the biobased RPUF prepared from these polyols increased from 19.0 vol.% to 22.6 vol.%, the improvement in the flame retardancy was not significant. This can be attributed to the low phosphorus content in long-chain biobased polyols.

In another approach, flame-retardant polyols with short chains are utilized for the synthesis and application of RPUF due to their elevated phosphorus content. Zou et al. [56] developed a hard-segment flame retardant (HSFR) to enhance the fire resistance of RPUF. By incorporating 13.8 wt.% of THPO, the resulting system was able to achieve a UL-94 V-0 rating, with an LOI ranging from 17.0 to 25.5%. During combustion, the HSFR can produce PO• and PO_2_• radicals in the vapor phase, which then react with flammable free radicals and impede segment decomposition. In the study conducted by Hu et al. [40], a flame-retardant polyol with a substantial phosphorus content was synthesized through a dehydrochlorination reaction. Hu et al. [57] synthesized a flame-retardant polyol with a high phosphorus content by performing a dehydrochlorination reaction. The addition of expandable graphite (EG) to the RPUF/BHPP system significantly enhanced the flame-retardant characteristics of the RPUF composites, achieving a high LOI value of 30.0%.

To achieve highly flame-retardant RPUF, Wang et al. [58] successfully synthesized a short-chain flame-retardant polyol. They placed particular emphasis on analyzing its compatibility with the conventional polyols 4110 and PEG400. The research findings demonstrated that, when these two mixed polyols were thoroughly ultrasonically blended to achieve full miscibility, their compatibility was significantly enhanced. This enhancement can impart RPUF with superior flame-retardant and mechanical properties.

### 2.2. Incorporation of Nitrogen-Containing Groups

The incorporation of nitrogen-containing groups is another effective approach for flame retardancy in polyurethane foam [59,60,61]. These compounds are usually incorporated into the polyol component during the production process, which then react with isocyanates to form polyurethane foam with improved flame retardancy [62]. During combustion, these compounds release nonflammable gases, such as nitrogen or ammonia, when exposed to high temperatures, diluting the flammable gases and reducing the combustibility of the foam [63,64,65]. Nevertheless, the flame-retardant effectiveness of nitrogen-based compounds is typically inferior to that of phosphorus-containing ones due to their single flame-retardant function.

**Figure 3:**
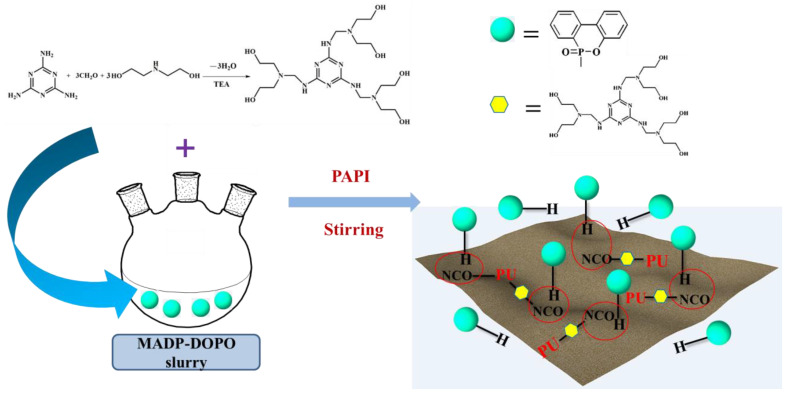
Schematic presentation of the interactions between PU−-NCO groups and DOPO [62] (Copyright 2018). Reproduced with permission from Elsevier Science Ltd.

**Figure 4:**
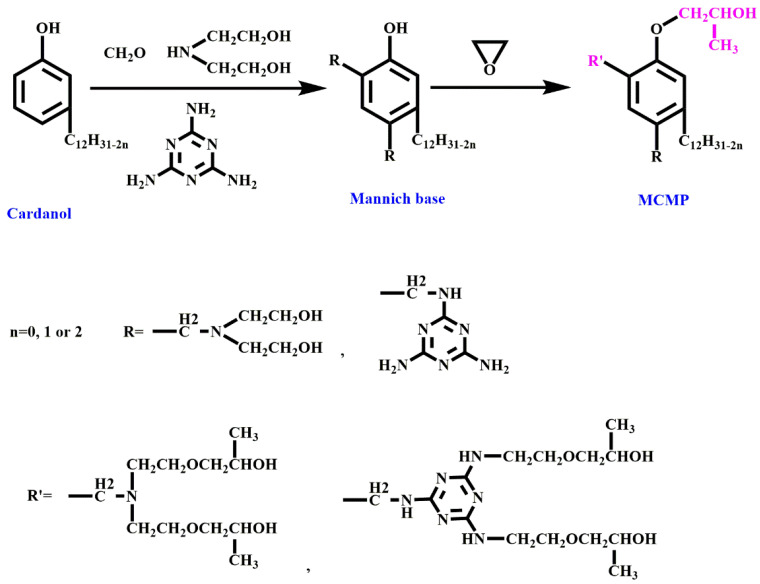
The synthesis of MCMP.

Melamine-based polyols are a type of reactive flame retardant used in the production of RPUF due to their high nitrogen content [66,67]. These polyols are synthesized by reacting melamine with an excess of formaldehyde and an alcohol or polyol, resulting in a highly crosslinked and thermally stable polymer. Hu et al. [62] developed a melamine-based polyol (MADP, Figure 2b) and incorporated it into the RPUF matrix, and the interactions between the PU-NCO groups and DOPO are shown in Figure 3. The findings from their study demonstrated a notable improvement in the LOI values (increasing from 19.0% to 28.5%) with the implementation of the flame-retardant system. This enhancement promotes the development of protective char layers and reduces the concentration of flammable gases in the gaseous phase. The synthesis of a Mannich base polyol derived from cardanol (MCMP, Figure 4) was conducted by Zhang et al. [68]. The incorporation of melamine into the molecular structure of MCMP led to improvements in the mechanical properties, thermal stability, and flame resistance of the resulting RPUF. Li et al. [69] developed an ecofriendly melamine-based polyether polyol referred to as GPP. They found that incorporating GPP in RPUF synthesis greatly enhances the flame retardancy of the resulting foam. The compressive strength of the RPUF samples increased by 106.0%, and an LOI value of 30.4% was achieved by fully incorporating GPP during the preparation process.

### 2.3. Incorporation of Sulfur-Containing Groups

Sulfur-based flame retardants represent an alternative category of reactive flame retardants employed in the manufacture of polyurethane foam. These compounds have the advantages of having a low cost, being effective, and having a low impact on the mechanical properties of the foam. A sulfur-containing polyol was synthesized by Bhoyate et al. [49] to create flame-retardant polyurethane foams with improved compressive strength without affecting the foam morphology or closed cell content. The modified RPUF exhibited a self-extinguishing time that was decreased from 94.0 s to 1.7 s when the phosphorus content reached 1.5 wt.% in contrast to the pristine RPUF.

## 3. Additive-Type Flame Retardants

Additive-type flame retardants for rigid polyurethane foam are typically nonreactive compounds that are added directly to the foam formulation to enhance its thermal stability and flame retardancy [27,28,70,71,72]. They can be either halogenated or nonhalogenated and can be divided into various subcategories based on their chemical composition, such as phosphorus-based, nitrogen-based, or metal-based flame retardants [73]. Additive flame retardants have various benefits compared to reactive flame retardants, such as their ease of integration into foam formulations and lower costs. Nevertheless, their constrained interfacial compatibility with the matrix results in a decline in the mechanical strength and thermal conductivity of the foam [74]. Furthermore, they may present certain disadvantages, such as potential migration from the matrix, which can influence the mechanical and thermal characteristics of RPUF, in addition to environmental concerns linked to their utilization.

### 3.1. Addition of Phosphorous-Containing Flame Retardants

The addition of phosphorous-containing flame retardants (P-FRs) in rigid polyurethane foam is a common approach to enhance its fire resistance. Phosphorous-containing compounds are considered effective flame retardants due to their ability to promote char formation in the condensed phase and to decrease the release of flammable gases in the condensed phase during combustion [75]. Examples of commonly used P-FRs in rigid polyurethane foam include ammonium polyphosphate (APP), dimethyl methylphosphonate, red phosphorous, and resorcinol bis(diphenyl phosphate) [63,76]. These flame retardants can provide varying degrees of fire resistance depending on their chemical structure, loading level, and processing conditions. It has been observed that the effectiveness of flame retardancy can be influenced by the valence state of phosphorus.

DOPO and its derivatives have recently emerged as P-FRs for the RPUF matrix, which release phosphorus species (PO•) and scavenge H• and OH• radicals in the flame to prevent the thermal degradation of polymers [77]. In a study by Zhang et al. [78], a new flame retardant called DOPO-BA was synthesized and added to a rosin-based RPUF matrix. When 20 wt.% DOPO-BA was incorporated, the LOI value increased from 20.1% to 28.1%. However, there was a significant reduction in the total smoke release.

Ranaweera et al. [79] addressed the migration issue of liquid flame-retardant dimethyl methylphosphonate (DMMP) in RPUF by synthesizing a biobased polyol from limonene and incorporating it with DMMP. They found that the addition of 2 wt.% DMMP can significantly reduce the burning time of RPUF by 83%. In addition, RPUF with TSPB exhibited improved water resistance. Wu et al. [80] observed that adding 10.6 wt.% toluidine spirocyclic pentaerythritol bisphosphonate (TSPB) to RPUF resulted in an improved LOI value and a UL-94 V-0 rating, which was attributed to the char-forming effect.

### 3.2. Addition of Phosphorus–Nitrogen-Based Flame Retardants

Nitrogen-containing flame retardants (N-FRs) are another class of commonly used flame retardants in the RPUF matrix that work by releasing nitrogen species in the gas phase during combustion, which can act as diluents fuels, oxygen, and free radicals in the flame [59]. The most used N-FRs in RPUF include melamine, melamine cyanurate, melamine phosphate, and guanidine derivatives. These N-FRs can provide excellent an flame retardancy performance in RPUF, especially when used in combination with phosphorus-containing flame retardants. The addition of N-FRs can also improve other properties of RPUF, such as its mechanical properties and thermal stability, while minimizing the smoke and toxic gas released during combustion. Xu et al. [81] investigated the smoke suppression mechanism of melamine in rigid polyurethane foam using a smoke density chamber, cone calorimetry, and a Py/GC-MS analysis.

**Figure 5:**
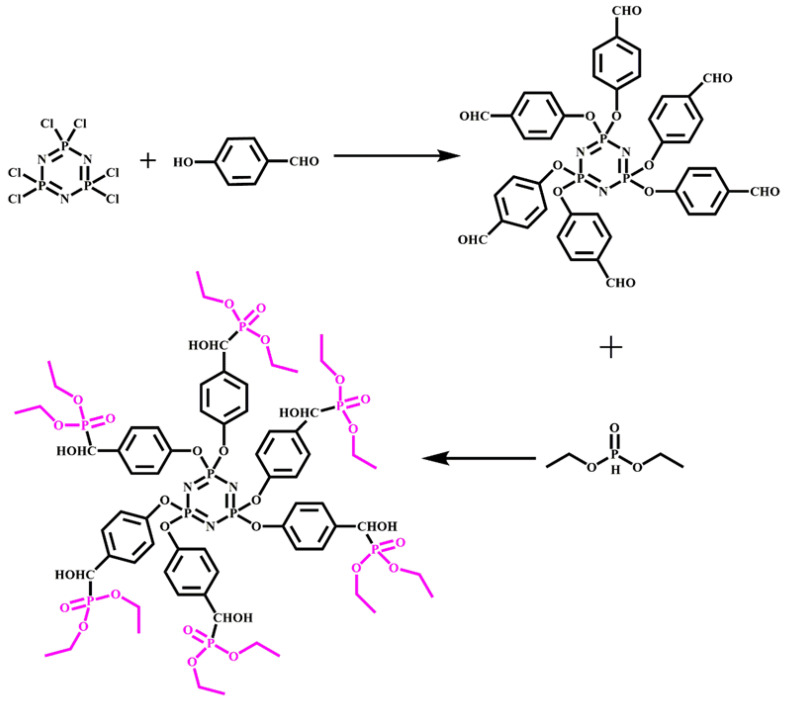
The synthesis of reactive flame-retardant HPHPCP.

A new phosphorus–nitrogen intumescent flame retardant (DPPM) was synthesized by Guo et al. [32]. The addition of only 9% DPPM was sufficient to enable RPUF to achieve a UL-94 V-0 rating and a limit oxygen index of 29%. Hexa(phosphitehydroxylmethylphenoxyl) cyclotriphosphazene (HPHPCP, depicted in Figure 5) is another flame retardant that has been successfully synthesized and incorporated into rigid polyurethane foam [32]. HPHPCP contains multifunctional groups that introduce crosslinking into the foam structure, thereby improving its thermal stability and compressive strength. The addition of HPHPCP to DPPM-RPUF resulted in an LOI of 29.5% and a UL-94 V-0 rating, which can be attributed to the surface pyrolysis of RPUF.

### 3.3. Addition of Expandable Graphite and Derivatives

**Figure 6:**
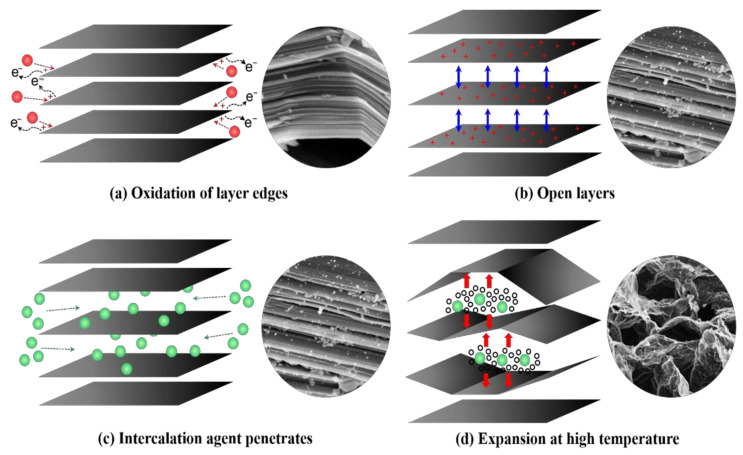
Preparation and expansion processes of expandable graphite [85] (Copyright 2019). Reproduced with permission from Elsevier Science Ltd.

Expandable graphite (EG) and derivatives have emerged as a promising flame retardant for rigid polyurethane foam (RPUF) due to their exceptional fire-resistant properties [82,83,84]. Expandable graphite (EG) is produced by introducing sulfuric acid, acetic acid, or nitric acid into the crystalline structure of graphite. This process results in a unique material with exceptional thermal expansion properties when exposed to heat (Figure 6) [85]. As a result, EG effectively decreases the flammability and heat release of RPUF. Wang et al. [86] employed a heterocoagulating method to encapsulate EG using magnesium hydroxide (MH). The incorporation of 11.5 wt.% core-shell EG@MH increased the limiting oxygen index (LOI) of the rigid polyurethane foam (RPUF) to 32.6% and improved the storage modulus by approximately 55.0%.

When considering the utilization of RPUF in refrigerators, it is essential that the material exhibits a thermal conductivity falling within the range of 19 to 22 mW/(m K) and a compressive strength exceeding 110 kPa. Akdogan et al. [87] developed a flame-retardant RPUF using 15 wt.% EG and 5 wt.% ammonium pentaborate (APB). The outcomes revealed that this RPUF exhibited a remarkable 42.8% reduction in the THR and a 77.0% decrease in the TSR compared to the pristine foam. Furthermore, it demonstrated an LOI of 27.9% coupled with a low thermal conductivity of 20.41 mW/(m K) and a high compressive strength of approximately 125 kPa, thus displaying promising characteristics for its potential utilization in refrigeration applications.

### 3.4. Addition of Nanoclay and Other Nanoparticles

Adding nanoclay and other nanoparticles to both flexible and rigid polyurethane foam is a promising strategy for decreasing the production of smoke particles and toxic gases during combustion [88,89,90]. Clay nanosheets are frequently used as nanoparticle fillers in polymer nanocomposites because of their low cost, widespread availability, and flexibility. Incorporating nanoclay (a two-dimensional nanoparticle) into rigid polyurethane foam can form a physical barrier that obstructs gas diffusion and heat transfer, resulting in reduced smoke production and increased thermal stability [34,35,91]. Furthermore, the platelet structure of nanoclay is attributed to the significant enhancement in the mechanical properties of RPUF, such as its strength, stiffness, and toughness [92]. Adilah Alis et al. [93] conducted a study on the flame retardancy and thermal stability of clay nanosheets, where halloysite nanotubes (HNTs) were synthesized and added to biobased RPUF. The study showed that increasing the HNT load from 1 wt.% to 5 wt.% led to a corresponding increase in the thermal stability of the RPUF. Moreover, RPUF blended with 5 wt.% HNTs exhibited the highest residual char of 18.1% at 600 °C compared to pristine RPUF with only 7.6%, indicating that the addition of HNTs significantly improved the char-forming ability of RPUF.

Similarly, nanoparticles, such as cuprous oxide (Cu_2_O), titanium dioxide (TiO_2_), nickel oxide (NiO), and silica (SiO_2_), have also been investigated for their potential in reducing smoke production and toxic gas emissions during rigid polyurethane foam’s combustion [94,95,96]. These nanoparticles act as flame retardants by catalyzing the formation of integral and compact chars, reducing the production of volatile compounds, and increasing the thermal stability of RPUF. A comparative study was conducted by Hu et al. [97] to investigate highly efficient catalysts for reducing toxic gas generation in RPUF nanocomposites at various temperatures. The primary objective of the study was to determine the most effective catalyst for minimizing the release of harmful gases during the combustion of RPUF. The findings indicated that both NiO and NiMoO_4_ were efficient catalysts in reducing toxic gas emissions. Furthermore, a comprehensive quantitative analysis of the gaseous degradation products, such as HCN, NO_x_, and CO, was performed for various polyurethane composite materials, including PU/Cu_2_O, PU/NiO, PU/MoO_3_, PU/CuMoO_4_, and PU/NiMoO_4_. This analysis was conducted using a tubular furnace method at both 650 °C and 850 °C.

**Figure 7:**
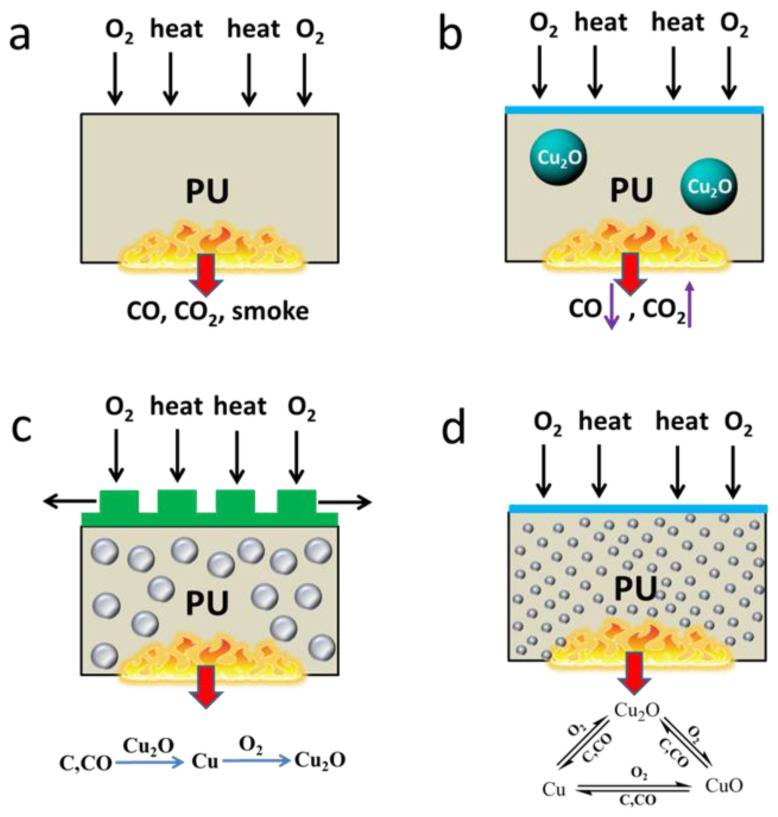
Schematic illustration for the mechanism of catalytic oxidation and catalytic carbonization of pristine RPUF (**a**), RPUF/Cu_2_O-1μm (**b**), RPUF/Cu_2_O-100nm (**c**) and RPUF/Cu_2_O-100nm (**d**) [98] (Copyright 2021). Reproduced with permission from Elsevier Science Ltd.

Yuan et al. [98] utilized a straightforward wet chemical method to synthesize cuprous oxide (Cu_2_O) crystals of varying sizes and investigated their impact on the combustion performance of RPUF. According to the study, the addition of 2 wt.% Cu_2_O to the RPUF matrix resulted in a notable decrease in the peak rate of carbon monoxide (CO) production and total smoke production. Furthermore, as illustrated in Figure 7, the reduction of Cu^2+^-Cu^+^-Cu^0^ by degraded gases and the oxidation of Cu^0^-Cu^+^-Cu^2+^ by oxygen were involved in the conversion of CO to CO_2_ and the complete combustion of RPUF.

### 3.5. Addition of Phase-Change Materials

The addition of phase-change materials (PCMs) to flame-retardant RPUF has been studied as a potential approach to enhance the thermal energy storage and fire retardancy properties of the material [99]. PCMs can absorb and release thermal energy during phase transition, thereby reducing temperature fluctuations and enhancing the thermal stability [100]. Several studies have investigated the effects of different types and concentrations of PCMs on the thermal and fire performance of RPUF, including the reduction in the peak heat release rate, total heat release, and smoke toxicity. The incorporation of PCMs in RPUF has the potential to be used in various applications, such as thermal insulation for buildings, refrigeration and thermal energy storage systems, and transportation industries. Niu et al. [101] focused on embedding a flame-retardant carbon nanotube (d-CNT) modified by DOPO into MPCMs to enhance their thermal stability and flame retardancy. The small room model and infrared thermal imager results also demonstrated that RPUF/d-c-MPCM could make indoor temperature fluctuation gentler, with a maximum indoor temperature of only 26.6 °C. Overall, the addition of PCMs to RPUF shows promise as a way to enhance both the thermal energy storage and fire-retardancy properties of the material, potentially leading to improved energy efficiency and safety in various applications.

## 4. Flame-Retardant Coatings

Flame-retardant coatings are a popular method to enhance the fire resistance of PUF. These coatings can be applied to the surface of PUF to create a protective layer that slows down the spread of fire and reduces the amount of heat and smoke released during combustion [102,103]. The coatings are typically made up of flame-retardant additives, such as aerogels, alumina trihydrate, metal hydroxides, and other inorganic materials that have a low flammability and good thermal stability. These additives work by releasing water vapor when exposed to heat, which helps cool down the surface of the foam and prevent it from igniting. In addition to enhancing the fire resistance of RPUF, flame-retardant coatings can also improve other properties, such as mechanical strength, chemical resistance, and UV stability.

**Figure 8:**
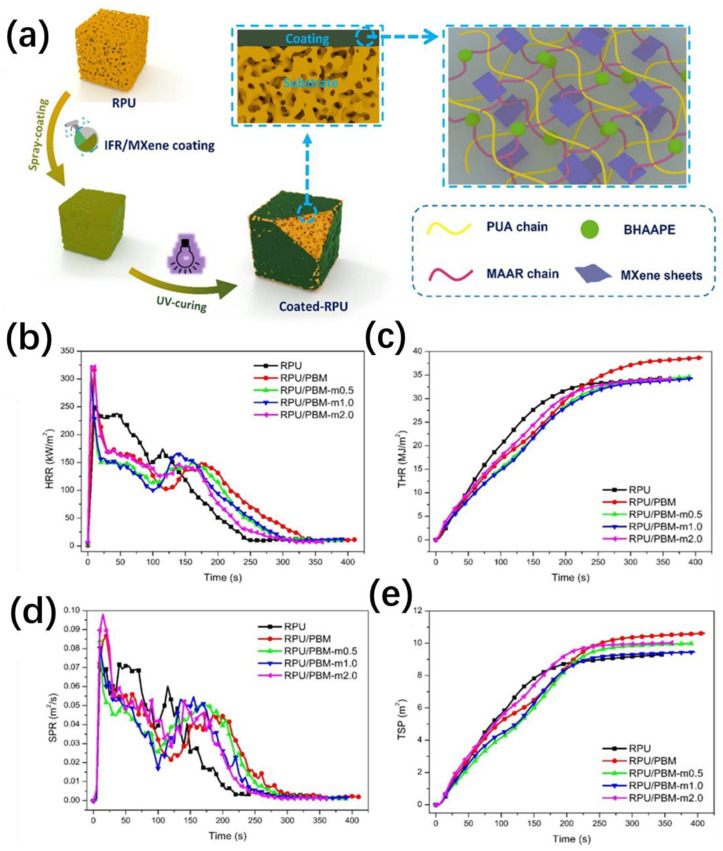
(**a**) Schematic illustration of the fabrication procedure of IFR/MXene-coated RPUF and (**b**) HRR, (**c**) THR, (**d**) SPR, and (**e**) TSP for RPUF and coated RPUF [105] (Copyright 2019). Reproduced with permission from Elsevier Science Ltd.

One example of a flame-retardant coating is the intumescent coating, which can form a char layer when exposed to fire, leading to reduced heat transfer and flame spread. Wang et al. [104] conducted a study in which they developed an intumescent coating by combining a silicone resin (poly-DDPM) and expandable graphite (EG). The resulting poly-DDPM/EG coating tightly adhered to the surface of RPUF and exhibited excellent flame retardancy. Additionally, the compressive strength of the coated RPUF increased significantly, up to 10%. As shown in Figure 8, Huang et al. [105] utilized UV-curable intumescent coatings to treat RPUF. By employing a spray-coating method, they applied a conformal IFR/MXene coating to the foam, aiming to enhance its fire safety.

**Figure 9:**
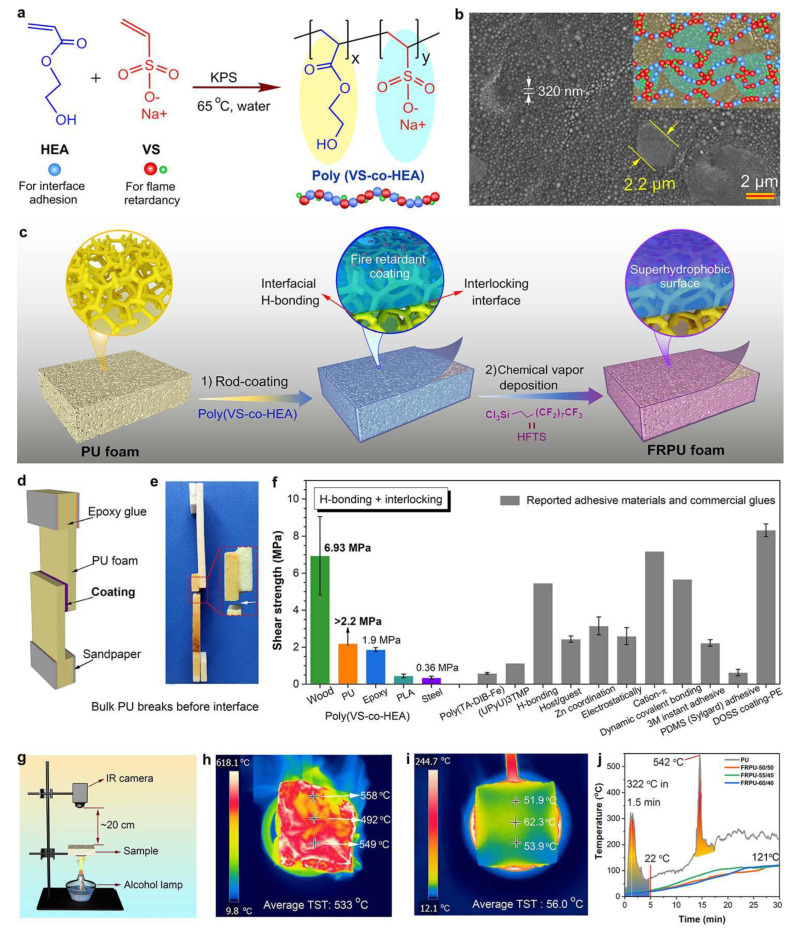
Preparation of RPUF with poly(VS-co-HEA) coatings [2] (Copyright 2021). Reproduced with permission from American Chemical Society. (**a**) Synthesis of bioinspired flame retardantpoly(VS-co-HEA) coatings and (**b**) a typical phase-separated micro/nanostructure of poly(VS50-co-HEA50). (**c**) Schematic illustration for the preparation process of flame-retardant rigid PU foam (FRPU). (**d**) The chart illustrating the adhesion or shear strength tests for PU foam. (**e**) Digital image of poly(VS-co-HEA) coatings against PU foam after shear tests, during which bulk PU foam broke before interfaces. (**f**) Shear strength of poly(VS-co-HEA) coatings against different substrates in comparison to some previous and commercial adhesives. (**g**) The homemade setup for determining the flammability of PU foam. The top-surface temperature (TST) determined by the IR camera for (**h**) untreated PU and (**i**) FRPU-60/40–600 μm after ignited for 15 min above an alcohol lamp. The sample thickness is ∼3.0 cm. (**j**) TSTs of PU and FRPU as a function of burning time.

In addition to intumescent coatings, other types of flame-retardant coatings have also been investigated for their effectiveness in enhancing the flame retardancy of RPUF. For example, a study by Chen et al. [45] reported on the development of a flame-retardant coating using alginate/clay aerogel. The coating was shown to reduce the flame spread rate, the HRR, and the THR of the RPUF during combustion as well as inhibit smoke production. The study showed that the facile and inexpensive posttreatment is a promising approach to improve the thermal stability and flame retardancy of RPUF. As depicted in Figure 9, motivated by the interfacial mechanical interlocking and hydrogen-bonding mechanisms observed in tree frogs and snails, Song et al. [2] successfully produced bioinspired flame-retardant poly(VS-co-HEA) coatings through a free-radical copolymerization process (refer to Figure 9). The thoughtfully engineered microphase-separated micro/nanostructure granted these coatings robust interfacial adhesion to the matrix [106]. The resulting RPUF exhibited notable fire-retardant properties, including a UL-94 V-0 rating and an LOI of 35.5%. Additionally, the treated foam showcased significant reductions in the peak heat release rate (PHRR) by 87.0% and total smoke release (TSR) by 71.0% compared to the untreated foam.

## 5. Concluding Remarks and Future Aspects

The primary objective of this review is to investigate the impact of reactive-type and additive-type flame retardants as well as flame-retardant coatings on the flame retardancy, mechanical properties, and smoke toxicity reduction of rigid polyurethane foam (RPUF). While the techniques discussed in this review have demonstrated favorable outcomes in enhancing the flame retardancy and thermal stability of RPUF, there remain several obstacles to overcome. These include issues such as additive migration, the inadequate interfacial compatibility between additive flame retardants and the RPUF matrix, the harmfulness of some flame-retardant agents, and the challenge of simultaneously achieving flame retardancy and mechanical properties. Furthermore, the inadequate interfacial compatibility between additive flame retardants and the matrix results in the degradation of the mechanical properties and thermal conductivity [107]. One potential opportunity for the future is the development of novel flame-retardant coatings for RPUF. These coatings could be made using sustainable and ecofriendly materials, such as cellulose-based materials, and could offer a promising alternative to conventional coatings. In addition, they could potentially enhance other desirable properties of RPUF, including its mechanical strength and insulation. Hence, one of the future directions in advancing flame-retardant RPUF involves utilizing functionalized precursors capable of yielding inherently flame-retardant foam while preserving the foaming process and physicochemical characteristics. Additionally, efforts should be made towards the development of biodegradable RPUF with flame-retardant properties to promote sustainability and wider applications.

## Data Availability

Not applicable.
